# Invasive Assessment of Coronary Microvascular Function

**DOI:** 10.3390/jcm11010228

**Published:** 2021-12-31

**Authors:** Fabio Mangiacapra, Michele Mattia Viscusi, Giuseppe Verolino, Luca Paolucci, Annunziata Nusca, Rosetta Melfi, Gian Paolo Ussia, Francesco Grigioni

**Affiliations:** Unit of Cardiovascular Science, Department of Medicine, Campus Bio-Medico University, Via Álvaro del Portillo, 200, 00128 Rome, Italy; m.viscusi@unicampus.it (M.M.V.); g.verolino@unicampus.it (G.V.); l.paolucci@unicampus.it (L.P.); a.nusca@unicampus.it (A.N.); r.melfi@unicampus.it (R.M.); g.ussia@unicampus.it (G.P.U.); f.grigioni@unicampus.it (F.G.)

**Keywords:** coronary physiology, coronary atherosclerosis, microvascular function

## Abstract

The critical role of the coronary microvascular compartment and its invasive functional assessment has become apparent in light of the significant proportion of patients presenting signs and symptoms of myocardial ischemia, despite the absence of epicardial disease, or after the adequate treatment of it. However, coronary microvascular dysfunction (CMD) represents a diagnostic challenge because of the small dimensions of the coronary microvasculature, which prevents direct angiographic visualization. Several diagnostic tools are now available for the invasive assessment of the coronary microvascular function, which, in association with the physiological indices used to investigate the epicardial department, may provide a comprehensive evaluation of the coronary circulation as a whole. Recent evidence suggests that the physiology-guided management of CMD, although apparently costly and time-consuming, may offer a net clinical benefit in terms of symptom improvement among patients with angina and ischemic heart disease. However, despite the results of several observational studies, the prognostic effect of the physiology-driven management of CMD within this population is currently a matter of debate, and therefore represents an unmet clinical need that urgently deserves further investigation.

## 1. Introduction

The invasive assessment of coronary artery disease (CAD) has historically focused on understanding the epicardial coronary anatomy and function. More sophisticated tools have been developed since it became clear that angiography was not sufficient to appropriately evaluate the composition and the ischemic potential of coronary atherosclerotic plaques. Intravascular imaging techniques and intracoronary physiology tests are now widely used to aid in diagnosing and managing CAD. However, there is much more to coronary circulation beyond epicardial arteries, including the microvasculature, which is often neglected, and which still represents a “black box” for clinical and interventional cardiologists. Coronary microcirculation is a complex and structured system of small vessels (caliber < 400 μm), which adapt their function in order to sustain the myocardium’s physiological needs. The clinical importance of this vascular compartment has become apparent in light of the significant proportion of patients presenting signs and symptoms of myocardial ischemia, despite the absence of epicardial disease, or even after the adequate treatment of it [1]. These patients are often labeled as affected by coronary microvascular dysfunction (CMD), although this condition is not explicitly diagnosed in most cases. CMD represents, in fact, a diagnostic challenge because of the small dimensions of the coronary microvasculature, which prevents direct visualization in vivo. This review provides an overview of the invasive techniques used to assess the coronary microcirculation and diagnose CMD.

## 2. Coronary Microvascular Dysfunction (CMD)

### 2.1. Phenotypes and Pathophysiological Mechanisms

Within the definition of CMD (Table 1), two major phenotypes of microvascular disease are currently included, each with a distinct underlying pathophysiological mechanism. Impaired endothelium-dependent vasodilation, mainly affecting the coronary arterioles, promotes the so-called “functional” CMD: the progressive endothelial dysfunction leads to a remarkable deterioration of nitric oxide (NO) production and release, thus providing insufficient NO-mediated vasodilation [2], or even to the paradoxical vasoconstriction of arterioles under conditions of increased myocardial oxygen consumption [3]. However, the pathophysiological mechanisms underlying microvascular vasomotor abnormalities are not limited to endothelial dysfunction: in fact, a dynamic and complex interplay between endothelial dysfunction, vascular smooth muscle cell hyper-reactivity, and triggering factors (i.e., inflammation, oxidative stress, genetic factors) is currently considered the most reliable hypothesis to explain epicardial and microvascular vasospasms [4]. The net clinical consequence is developing symptoms (i.e., microvascular angina) and the electrocardiogram (ECG) signs of myocardial ischemia. The resulting microvascular spasm has been described as one of the most prevalent mechanisms of CMD among patients with ischemia and nonobstructive coronary arteries (INOCA) [5]; however, its epidemiological burden in the overall population is still unknown. When microvascular spasm is suspected, the diagnostic approach offering the highest efficacy-safety profile is the acetylcholine test [6]: The administration of high doses of intracoronary boluses of acetylcholine (2–100 μg), acting on both the epicardial and microvascular districts, enables the unmasking of any underlying vasomotor abnormalities. According to the Coronary Vasomotion Disorders International Study Group (COVADIS) criteria [7], the vasoreactivity test meets the criteria for microvascular vasospasm when it reproduces the symptoms usually experienced by the patients, and when it triggers ischemic ECG changes in the absence of a significative epicardial spasm (<90% coronary diameter reduction).

Nevertheless, several other non-endothelium-dependent mechanisms may equally lead to CMD. In particular, the microvascular wall remodeling, due to the increased wall-to-lumen ratio (intimal thickening and perivascular fibrosis), with or without the loss of the myocardial capillary density (capillary rarefaction), may cause a decrease in the microcirculatory conductance, with an impaired oxygen delivery capacity [8,9]. Of note, this structural phenotype of CMD shows the specific hemodynamic features of microvascular dysfunction, such as an impaired blood flow across the epicardial and microvascular coronary vessels (invasively defined as a decline in the coronary flow reserve), and increased coronary microvascular resistance, even during drug-mediated hyperemia. Interestingly, when structural and functional CMDs concur, the invasive evaluation reveals signs of both an impaired microvascular vasomotor tone, and structural microcirculatory remodeling. However, on the basis of our current knowledge, none of the available diagnostic tools are able to definitely and precisely discriminate between the different phenotypes of CMD. In fact, according to the results of both adenosine and provocative tests, we may theoretically distinguish between structural and functional CMD. By contrast, in daily clinical practice, a frequent overlap of those phenotypes occurs, and the exact pathophysiological contribution of each mechanism is still challenging to establish [10].

### 2.2. Clinical Presentation

Irrespective of the underlying pathophysiological mechanism, CMD seems to be associated with traditional cardiovascular risk factors. A large body of evidence highlights a higher incidence of CMD among females who smoke (particularly following menopause, when the dysregulation of estrogenic homeostasis may deteriorate coronary microvascular function) [11], or among patients affected by diabetes mellitus [12], hypertension [13], or dyslipidemia [14,15], which suggests that these conditions may have a central role in the development of both endothelial dysfunction and coronary wall rearrangement. Moreover, the higher prevalence of CMD in noncardiac inflammatory disease, such as rheumatoid arthritis or systemic lupus erythematosus [16], proves that various and currently not established inflammatory mechanisms may be involved in the progression of CMD.

Microvascular angina (MVA) represents the more specific and reliable symptom of a broad and heterogeneous spectrum of disorders known as INOCA (ischemia with nonobstructive coronary arteries), of which CMD is a relevant component. According to the COVADIS definition [7] (Table 2), angina meets the diagnostic criteria for MVA if the following requirements are satisfied: (1) Symptoms of myocardial ischemia (classic effort/rest angina, or angina equivalent); (2) The absence of obstructive CAD (a <50% diameter reduction or a fractional flow reserve (FFR) > 0.80), assessed invasively or noninvasively; (3) Objective evidence of myocardial ischemia (spontaneous or stress-induced ECG changes, myocardial perfusion, and/or wall motion abnormalities); (4) Evidence of impaired coronary microvascular function.

Among the causes of INOCA, vasomotor tone abnormalities of both the epicardial and the microvascular districts play a crucial role. The sudden occurrence of chest pain due to coronary spasm, so-called “vasospastic angina” (VSA), should be suspected according to the COVADIS criteria [7] if the following criteria are fulfilled (Table 2): (1) Nitrate-responsive angina; (2) Transient ischemic ECG changes; and (3) Coronary artery spasm, defined as a transient total, or subtotal, coronary artery occlusion (>90% constriction), either spontaneously, or in response to a provocative stimulus. However, it should be highlighted that the absence of a clear epicardial vasospasm within the context of arguable vasomotor tone abnormalities may raise the suspicion of a prevalent microvascular spasm, particularly if the invasive physiological assessment reveals an impaired blood flow across the coronary vessels.

## 3. Coronary Flow Reserve (CFR)

**Figure 1:**
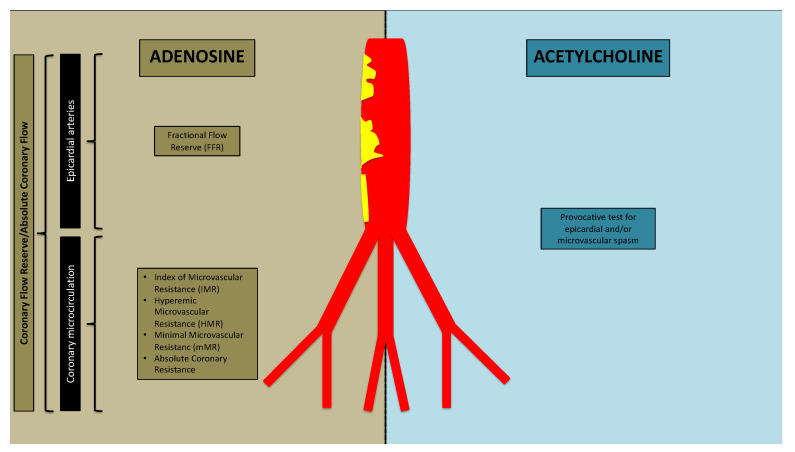
Invasive assessment of coronary circulation.

Blood flow across coronary vessels consists of a highly dynamic phenomenon, aimed at delivering an adequate blood supply to the myocardium, either at rest or during exercise. The ability of the entire coronary bed (both epicardial and microvascular districts) to actively adapt its size to satisfy any increased myocardial oxygen demand is invasively and noninvasively assessed with a physiological index known as the “coronary flow reserve” (CFR, Figure 1). Although it has been initially employed for research purposes, the growing body of evidence supporting the prognostic impact of CMD, especially in the absence of epicardial flow-limiting stenosis, has progressively promoted its adoption in clinical practice in order to systematically ensure a comprehensive evaluation of the coronary physiology, particularly in patients with ischemic heart disease and a low-to-intermediate degree of epicardial coronary stenosis. CFR measurement allows for the appraisal of the epicardial and microvascular functions together, thus providing additional information for patients with chest pain and an absence of critical CAD.

The CFR may be measured invasively using thermodilution or Doppler flow velocity (Table 3), or noninvasively, with transthoracic Doppler echocardiography, as well as with positron emission tomography (PET), and stress cardiac magnetic resonance (CMR). The adoption of a Doppler wire (ComboWire XT or FloWire, Philips Volcano Corporation, San Diego, CA, USA) empowers an assessment of the CFR at rest, and after hyperemic stimuli (140 mg/kg/min of intravenous adenosine [17]), as the ratio of the hyperemic to the resting coronary flow velocity (CFV): $\frac{CFVhyper}{CFV\;rest}$ [18]. The measurement of the Doppler velocity of the coronary flow may also be performed noninvasively with transthoracic echocardiography [19,20,21]; in fact, the visualization of the left anterior descending artery in the parasternal short-axis view usually allows for the measurement of the CFV at rest and after hyperemia, thus indirectly providing the CFR value. However, noninvasive estimates of the CFR with transthoracic echocardiography do not offer a complete and detailed evaluation of the coronary flow since only the left anterior descending artery is conventionally used for CFV assessment, which represents the major technical limitation of this technique [22]. On the other hand, despite the thermodilution-derived measurement resulting in an adequately accurate and reliable CFR evaluation [23,24], there is currently no available evidence clearly supporting the adoption of either thermodilution-based or Doppler-derived methods, according to the most recent consensus statements. The adoption of a specific pressure wire (PressureWire XTM, Abbott Vascular, Santa Clara, CA, USA) with three sensors (proximal temperature, distal pressure, and distal temperature) enables the flow measurement through thermodilution: more specifically, three consecutive intracoronary saline injections at room temperature are conventionally performed, thus obtaining the interval of time needed for the saline bolus to travel from the tip of the guiding catheter to the distal temperature sensor of the pressure wire, which is known as the mean transit time (Tmn). After that, the coronary blood flow is estimated inversely proportional to the time it takes for an injected bolus of room temperature saline to travel down the coronary artery ($\frac{1}{Tmn}$), and it can be easily derived from the coronary thermodilution curve. It is worth noting that a strong correlation between the thermodilution-derived coronary flow index and the true coronary flow was found [25,26], thus encouraging its adoption as a reliable and safe technique with which to assess the CFR as the resting Tmn divided by the hyperemic Tmn (CFR = $\frac{Tmn\;baseline}{Tmn\;hyperemic}$). With regard to invasive methods, the reasonable CFR cut-off values showing a significant prognostic impact are <2.0 for thermodilution-based measurements [27,28], and <2.5 for Doppler-based measurements [29,30].

Of note, several other noninvasive methods (PET and CMR) used for assessing the CFR have been considered with encouraging results: both PET and CMR seem to provide independent prognostic information in patients with known or suspected CAD [31,32,33]. At last, another indirect marker of suspected impaired coronary flow has been studied: the corrected TIMI frame count (CTFC). This is an objective and quantitative method that uses the number of cine frames needed for the dye to run off from the coronary artery as an indirect index of the coronary flow [34]. A CTFC > 27 frames indicates delayed contrast run-off and, consequently, a possible microvascular disease [35].

A comprehensive assessment of coronary circulation concerning the functional relevance of epicardial CAD using hyperemic and non-hyperemic indexes and the CFR grants a detailed analysis of ischemic heart disease (Figure 1). More specifically, several combinations of epicardial and microvascular disease are available, according to the coronary physiology indexes [36]: (1) Epicardial CAD with severe flow-limiting stenosis (FFR ≤ 0.80) and an impaired flow supply (CFR < 2), due to an inadequate drop of the microvascular resistance; (2) Epicardial CAD with severe flow-limiting stenosis (FFR ≤ 0.80) and a preserved flow supply (CFR ≥ 2); (3) Microvascular CAD or predominant diffuse epicardial atherosclerosis with an intact FFR (>0.80) and an impaired CFR (<2); (4) Nonischemic coronary lesions with both a preserved FFR (>80) and CFR (≥2). The prognostic impact of the discordant coronary physiology indexes has been largely debated [36,37]. In particular, patients with severe flow-limiting stenosis (FFR ≤ 0.80) and a normal coronary flow (CFR ≥ 2) present worse outcomes than those with normal values of both measures (FFR > 0.80 and CFR ≥ 2) when treated medically, thus supporting the hypothesis that the FFR should primarily guide revascularization decisions, regardless of the CFR reading [38]. On the other hand, patients with a preserved FFR (>0.80) but a reduced CFR (<2) have been shown to experience a higher incidence of unfavorable outcomes compared to those with a preserved FFR and CFR, thus highlighting the critical prognostic role of coronary microvascular disease in ischemia-driven adverse events [30].

## 4. Index of Microcirculatory Resistance (IMR)

According to Ohm’s law, the vascular resistance (R) is equal to the driving pressure (∆P) divided by the flow rate (Q): R = ∆P/Q. ∆P is the pressure difference across the myocardium (Pd – Pv), and Q represents the coronary flow, which is known to be inversely related to the Tmn ($\frac{1}{Tmn}$). Therefore, coronary microvascular resistance (R) can be calculated as follows: Pd − Pv/$\frac{1}{Tmn}$ = (Pd − Pv) × Tmn.

Assuming that the venous pressure is close to zero (Pv = 0), the final equation will be: Pd × Tmn. Therefore, the index of coronary microvascular resistance (IMR) is calculated with thermodilution as the product of the distal coronary pressures (Pd) and the Tmn during maximal hyperemia (Table 3) [39]. Of note, a strong correlation between the thermodilution-derived coronary microvascular resistance index (IMR) and the true microcirculatory resistance (TMR) was found. In particular, IMR values ≥ 25 suggest high microvascular resistance and, indirectly, coronary microvascular dysfunction [40].

Although the IMR is independent of the hemodynamic state and the coronary flow, many studies have shown a relevant overestimation of the microvascular resistance with critical epicardial stenosis. In the presence of severe epicardial stenosis, both the coronary flow and the collateral flow contribute to the overall myocardial flow; therefore, assuming the IMR as the product of the Pd and the Tmn during maximal hyperemia, one or more epicardial flow-limiting stenoses may significantly prolong the time needed to travel down the coronary artery, thus determining a higher IMR [41]. However, when the collateral flow is taken into account by incorporating the coronary wedge pressure (Pw) into a more complex formula [Pa × Tmn Hyper × (Pd − Pw/Pa − Pw)], the IMR remains constant in both experimental models [42] and humans [43,44].

## 5. Hyperemic Microvascular Resistance (HMR)

Theoretically similar to the thermodilution-based IMR, hyperemic microvascular resistance (HMR–Figure 1) is a Doppler-derived technique for the invasive assessment of CMD, and it is defined as the ratio of the mean distal pressure (Pd) to the average peak flow velocity (APV) during maximal hyperemia (HMR = $\frac{Pd}{APV}$, Table 3). Some initial concerns have been raised since it may overestimate the actual microvascular resistance in the presence of a substantial collateral flow contribution due to critical epicardial stenosis. In fact, according to the equation mentioned above, HMR is inversely correlated to the Doppler-derived coronary flow, which is reduced across the epicardial vessels with functionally relevant CAD. However, after correcting for the collateral flow (Qc), the HMR (HMR = $\frac{Pd}{Qs+Qc}$) was equal to the actual microvascular resistance [45]. The HMR seems to carry a consistent prognostic significance, particularly after acute myocardial infarction (AMI). Within this cohort of patients, the HMR measured immediately after percutaneous coronary intervention (PCI) precisely identifies the patients with post-AMI microvascular injury with a higher risk of adverse clinical outcomes [46]. However, the cut-off values of HMR that confer the optimal prediction of CMD are still a matter of debate. A study enrolling patients with chest pain and the absence of angiographically significant coronary stenosis showed that the values of HMR ≥ 1.9 independently correlate with recurrent angina [47], while other studies propose a more stringent cut-off of ≥2.5 for CMD diagnosis [48].

Although a gold standard for coronary microvascular assessment has not yet been established, several studies have compared the thermodilution-derived IMR and the Doppler-derived HMR, aiming to determine the level of agreement between the IMR and the HMR, and to compare the ability of the IMR and the HMR to predict the independent measures of CMD. However, only a modest correlation was found between them, thus suggesting that these measurements cannot be considered equivalent predictors of CMD [48].

## 6. Other Hyperemic Methods of CMD Assessment

### 6.1. Minimal Microvascular Resistance (mMR)

Minimal microvascular resistance (mMR–Figure 1) is another Doppler-derived invasive method of coronary microvascular resistance assessment and is defined as the ratio between the distal coronary pressure and the flow velocity during the hyperemic wave-free period ($\frac{Pd}{Qs\left[wf\;period+hyper\right]}$, Table 3). It has been developed as a CMD index to overcome the previously mentioned limitations of both the IMR and the HMR in the presence of obstructive CAD. In this setting, the withdrawal of the collateral flow contribution leads the IMR and the HMR to overestimate the true microvascular resistance status. De Waard et al. show that the invasive assessment of coronary microvascular resistances within the diastolic wave-free period during maximal hyperemia enables a reliable evaluation of the coronary microcirculation status, irrespective of the epicardial flow [49]. For this reason, mMR has been proposed as a clinical measure of microvascular disease, in both ischemic and nonischemic cardiopathy.

### 6.2. Resistive Reserve Ratio (RRR)

The resistive reserve ratio (RRR) is a thermodilution-derived index that integrates both the coronary flow and the pressure as the ratio between the basal and hyperemic microcirculatory resistances ($\frac{BIR}{IMR}$, Table 3). As a consequence, this index highlights the ability of the coronary microcirculation to vary its resistance via vasodilation in response to adenosine. For instance, higher RRR values indicate a greater vasodilatation of the microcirculation in response to hyperemia, while lower RRR values indicate the poor vasodilator capacity of the coronary microcirculation.

The RRR has been initially investigated as a potential predictor of post-PCI complications in acute coronary syndromes (ACS). In a population of ST-segment elevation myocardial infarction (STEMI) patients, an RRR ≤ 1.7 was associated with the coronary microvascular obstruction (CMVO) extent, myocardial hemorrhage, the infarct size, and adverse clinical outcomes [50]. The prognostic impact of the RRR has also been investigated in stable CAD patients undergoing elective PCI. The RRR is confirmed to be a reliable predictor of long-term adverse clinical events in this setting, thus suggesting its adoption, in addition to other coronary physiology indexes, to improve risk stratification and to guide decision making [51].

## 7. Absolute Coronary Flow and Resistance

Both the CFR and the IMR are thermodilution-based physiological indexes in which the coronary flow and the microvascular resistances are indirectly estimated via the Tmn of a manually injected saline bolus, thus implying that its measurement depends, to a certain degree, on an operator injection technique. Moreover, both the CFR and the IMR require the achievement of adenosine-induced stable hyperemia.

The principle of absolute coronary flow (Q) and microvascular resistance (R) has been proposed to overcome these limitations through the adoption of a technique that requires a continuous infusion of saline and thermodilution [52]. Continuous thermodilution became applicable in catheterization laboratories since a dedicated monorail infusion catheter (RayFlow, Hexacath, Paris, France) and the appropriate software (CoroFlow, Coroventis, Uppsala, Sweden) became available in 2016. It presents several advantages over the traditional CFR and IMR measurements for the following reasons: (1) It has been demonstrated to be safe, reproducible, and operator-independent; (2) It does not require pharmacological-induced hyperemia since the continuous saline injection produces a prolonged and steady physiological hyperemic state within seconds (Table 3).

Both the absolute coronary flow and resistance (Figure 1) have been fully validated in humans [53]. However, their adoption in everyday clinical practice has been precluded since their “normal” values are still a matter of debate. A recent contribution by Fournier et al. found that, in normal individuals, hyperemic Q equals approximately 670 mL/min, whereas the total resistance of coronary circulation is approximately 150 WU [54]. In particular, stable maximal hyperemia may be obtained with a saline infusion at room temperature at a rate of 20 mL/min [55]. Moreover, a recent contribution by Gallinoro et al. found that the resting coronary blood flow and microvascular resistance may be assessed with infusion rates of 8-to-10 mL/min of saline, whereas the CFR and the microvascular resistance reserve may be directly estimated with infusion rates of 10-to-20 mL/min of saline, showing an excellent agreement with the Doppler wire-derived CFR, thus demonstrating, for the first time in humans, that continuous thermodilution enables an accurate assessment of the true quantitative flow and the resistance reserve measurements [56].

The main limitation of continuous thermodilution is that it does not provide enough information regarding the amount of myocardial mass subtended by the coronary flow. However, the association of continuous thermodilution with noninvasive methods of myocardial mass quantification (computed tomography or magnetic resonance imaging) has been proposed to compare the standardized values of either the CFR or the IMR from different myocardial territories and patients [57].

In order to outline a surrogate of microvascular function that is based on the operator-independent measurements of the absolute flow and pressures, as well as one that is unrelated to the epicardial resistance, the autoregulation principle, and the myocardial mass and, therefore, that is specific to coronary microcirculation, the microvascular resistance reserve (MRR) was proposed by De Bruyne et al. as a novel index of the microcirculatory function. In an analogy to the FFR concept, the MRR has been described as the ratio between the resting and hyperemic microvascular resistances, theoretically depicting a quantitative assessment of the extent of the potential microvascular resistance decrease resulting from a hyperemic stimulus. In its general form, the MRR can be expressed as the ratio of the CFR to the FFR, corrected for the driving pressures. However, the MRR cut-off values, as well as the clinical and prognostic relevance of the MRR compared to the other existing indexes of microvascular function, are not yet currently established [58].

Initial exploratory results about the potential clinical significance of both the absolute coronary flow and resistance have been reported by Konst et al. [59]: in a cohort of INOCA patients who underwent invasive physiological assessment (FFR, acetylcholine testing, and adenosine administration), the absolute coronary flow was the best predictor of self-reported angina, whereas the absolute microvascular resistance was unrelated to epicardial coronary vasospasm, but was significantly increased in INOCA patients with CMD. However, the exact predictive value and the prognostic impact of continuous thermodilution-derived indexes need to be further investigated in more extensive studies [60].

## 8. Clinical Utility of Microvascular Function Assessment and Future Perspectives

The invasive functional assessment of coronary microcirculation represents an essential resource for deeply understanding the broad spectrum of ischemic cardiopathy mechanisms and improving the treatment and management of these patients. Although CMD investigation has been initially restricted to cardiovascular research, several studies support the hypothesis of the potential clinical benefit of its adoption in daily practice, particularly in patients with CAD in the absence of flow-limiting epicardial stenosis [61]. The routine introduction of an invasive microcirculatory evaluation may bring the following advantages: (1) A more accurate diagnosis of the underlying pathophysiological mechanism of CAD, especially among INOCA patients; (2) Better risk stratification in order to avoid further unnecessary invasive procedures; and (3) More tailored management that accords with the specific mechanism of CAD. The CorMiCA trial [62] results were raised as a true game-changer in the ongoing debate about the potential feasibility of a comprehensive assessment of the coronary microvasculature function in everyday clinical practice. For the first time, in a randomized placebo-controlled blinded clinical trial, Ford and colleagues provided evidence of a net clinical benefit of physiology-guided management among a population of patients affected by stable CAD. Remarkably, within a population of INOCA patients, traditional invasive coronary angiography, combined with a comprehensive physiological assessment of both the epicardial and microvascular districts (FFR, CFR, IMR, and vasoreactivity tests) led to a better stratification of the INOCA endotypes, and, thus, to a more appropriate etiology-based therapeutic strategy. This example of stratified medicine substantially improved patient-oriented outcomes, which included reduced angina severity and better quality of life at six months of follow-up. Interestingly, the previous results were confirmed at one year of follow-up [63], thus supporting sustained symptom improvement. However, whether a routine invasive assessment of the coronary microvascular function can empower a prognostic impact on “hard” clinical endpoints or not is still a matter of debate. Therefore, more extensive randomized clinical trials explicitly addressing this issue are required.

Moreover, the prognostic impact of CMD among patients who underwent PCI has been largely investigated. When already present before the intervention, microvascular dysfunction may predict periprocedural outcomes in patients with stable CAD [64,65], thus supporting the hypothesis that an impaired baseline coronary microcirculatory reserve may reduce the ability to tolerate any further ischemic insults.

In addition, several pieces of evidence supporting the key prognostic role of the postprocedural abnormal IMR are currently available. High post-PCI microvascular resistances are significantly predictive of poor procedural-related and long-term outcomes for patients undergoing both elective [66,67] and primary PCI [68]. Multiple mechanisms contribute to postprocedural microvascular dysfunction in stable CAD, including plaque disruption, microvascular spasms, embolism, an enhanced inflammatory response, endothelial dysfunction, and platelet activation [69,70], whereas, in the acute setting, the high procedural-related thrombotic burden may produce prognostically relevant abnormalities of the coronary microcirculation, due to CMVO.

Coronary microcirculation has also been extensively assessed in the setting of an ACS. CMD and CMVO, a well-known complication of coronary interventions in the presence of a high thrombotic burden, occur in up to half of the patients undergoing otherwise successful primary PCI [71]. In fact, in a variable proportion of patients presenting with a STEMI ranging from 5% to 50%, primary PCI achieves epicardial coronary artery reperfusion but not myocardial reperfusion, a condition known as “no-reflow” [72]. This phenomenon is quantitatively described with the TIMI flow grade, a numeric index attributing a 0 to 3 score to the vessel opacification (0 = no flow, 3 = normal flow). Mechanisms underlying any degree of impaired flow after PCI vary, and include distal embolization, ischemia-reperfusion injury, and the individual predisposition of coronary microcirculation to injury [72]. The prognostic relevance of CMVO in ACS has been well established: patients with STEMI complicated by CMVO show a significantly higher incidence of death and rehospitalization for heart failure than those with optimal myocardial reperfusion [73]. Therefore, a comprehensive assessment of microvascular resistance seems to be a further legitimate method to identify the cohort of patients more susceptible to postprocedural microvascular injury. Moreover, even in the absence of evident CMVO, postprocedural microvascular resistances correlate with more extensive myocardial damage, and potentially with a higher incidence of adverse events at follow-up [74], thus suggesting that the IMR may play an essential role as an early diagnostic marker for CMD, as well as a measure of invasive treatment efficacy.

Several preventive measures have been investigated with regard to the wide range of mechanisms contributing to PCI-related microvascular dysfunction and myocardial injury. Nicorandil, a potent cardioprotective agent, which acts by selectively opening the mitochondrial adenosine triphosphate-dependent potassium channels, improves coronary perfusion in both the epicardial and microvascular compartments [75]. Interestingly, the administration of intravenous nicorandil during primary PCI for STEMI showed significant short-term and long-term benefits in cardiovascular event prevention [76], due to its crucial role in coronary microvascular vessel dilation.

Similar perioperative results have been observed after the periprocedural administration of verapamil. In particular, post-PCI improvements of the microvascular function appear to be reasonably attributable to the beneficial effect of intracoronary verapamil in preventing [77], or reversing [78], the no-reflow/slow-flow phenomenon.

The available evidence with regard to perioperative myocardial injury prevention measures have been further expanded with the ProMicro (PROtecting MICROcirculation during coronary angioplasty) study. In this randomized placebo-controlled clinical trial, administering a single bolus of enalaprilat before PCI improved coronary microvascular function, and protected the myocardium from procedure-related injury in patients undergoing elective PCI [74]. Among stable CAD patients, periprocedural myocardial injury due to microvascular impairment seems mainly driven by high platelet reactivity [69,79] and a transient endothelial dysfunction, related to an impaired platelet response to clopidogrel [80]. As a consequence, the hypothesis that an enhanced antiplatelet effect with a different P2Y12 inhibitor could exert a protective effect on the microcirculation during elective PCI has been explored in the ProMicro 2 trial [81]: notably, we demonstrated that, compared with clopidogrel, a single loading dose of prasugrel was able to prevent microvascular impairment and ischemic complications in the setting of elective PCI. Furthermore, a significant correlation was found between the IMR and platelet reactivity, thus confirming that the protective effect of prasugrel on coronary microcirculation is mainly attributable to the attenuation of the PCI-related increase in the platelet reactivity [82].

However, some contrasting evidence is currently available. In the randomized controlled Strategies of Loading With Prasugrel Versus Clopidogrel in PCI-Treated Biomarker Negative Angina (SASSICAIA) trial [83], a pre-PCI loading dose of prasugrel compared with clopidogrel resulted in a nonsignificant 69% relative decrease in the rate of type 4a AMI. In contrast, the ALPHEUS study found that ticagrelor was not superior to clopidogrel in reducing periprocedural myocardial necrosis after elective PCI [84]. However, in both these studies, the periprocedural coronary microcirculation status was not investigated, and, importantly, a sufficient platelet inhibition was likely not achieved at the time of intervention because of an inadequate delay from the P2Y12 loading dose to the PCI. Consequently, larger studies are required to assess the short- and long-term prognostic impacts of an adequate platelet inhibition on the postprocedural coronary microvascular function, as well as the safety of a more potent antiplatelet therapy.

## 9. Conclusions

Although appearing costly and time-consuming, the invasive microvascular assessment allows for the achievement of crucial information to establish more appropriate decision-making processes and therapeutic strategies for patients with ischemic heart disease. In fact, despite all of the recent research and clinical efforts, the global burden of myocardial ischemia persists at unacceptably high levels, resulting in a worse quality of life and a poorer prognosis [85], particularly in patients with ischemic signs and symptoms in the absence of evident epicardial disease, or with persistent ischemia after its adequate treatment. Moreover, these patients exemplify the conundrum of CMD, a puzzling clinical entity embodying several endotypes that occasionally overlap with each other. Currently, interventional cardiologists propose multiple different techniques for the invasive evaluation of the coronary microvascular district, which provide comprehensive physiological assessments. Recent evidence suggests that the physiology-guided management of CMD may offer a net clinical benefit in terms of symptom improvement among patients with angina and ischemic heart disease. However, despite the results of several observational studies, the prognostic effect of the physiology-driven management of CMD within this population is currently not established, and it therefore represents an unmet clinical need that urgently deserves further investigation, with more extensive randomized clinical trials.

## Figures and Tables

**Table 1:** Pathophysiological mechanisms and hemodynamic profiles of coronary microvascular dysfunction (CMD).

Pathophysiologic Mechanisms	Hemodynamic Profiles	
Coronary Microvascular Dysfunction Structural remodeling	Adenosine test CFR < 2.0 IMR ≥ 25 HMR > 1.9/HMR > 2.5	Acetylcholine test No ischemic symptoms and ECG changes No or <90% coronary diameter reduction
Coronary Microvascular Dysfunction Microvascular spasm	CFR < 2.0 IMR < 25 HMR ≤ 1.9/HMR ≤ 2.5	Ischemic symptoms and ECG changes No or <90% coronary diameter reduction
Coronary Microvascular Dysfunction Structural remodeling and microvascular spasm	CFR < 2.0 IMR ≥ 25 HMR > 1.9/HMR > 2.5	Ischemic symptoms and ECG changes No or <90% coronary diameter reduction
Coronary Epicardial Vasospasm	CFR < 2.0 IMR < 25 HMR ≤ 1.9/HMR ≤ 2.5	Ischemic symptoms and ECG changes coronary diameter reduction ≥ 90%
Coronary Microvascular Dysfunction and Epicardial Vasospasm	CFR < 2.0 IMR ≥ 25 HMR > 1.9/HMR > 2.5	Ischemic symptoms and ECG changes coronary diameter reduction ≥ 90%

CFR: coronary flow reserve; IMR: index of microvascular resistance; HMR: hyperemic myocardial velocity resistance.

**Table 2:** Diagnostic criteria of microvascular angina (MVA) and vasospastic angina (VSA), according to the COVADIS definition.

Microvascular Angina (MVA)	Vasospastic Angina (VSA)
**Symptoms of myocardial ischemia:** ◦ Effort and/or rest angina ◦ Angina equivalents (i.e., shortness of breath) **Absence of obstructive CAD** (<50% diameter reduction or FFR > 0.80) by: ◦ Coronary CTA ◦ Invasive coronary angiography **Objective evidence of myocardial ischemia:** ◦ Ischemic ECG changes during an episode of chest pain ◦ Stress-induced chest pain and/or ischemic ECG changes in the presence or absence of transient/reversible abnormal myocardial perfusion and/or wall motion abnormality **Evidence of impaired coronary microvascular function:** ◦ Impaired coronary flow reserve (cut-off values, depending on methodology use, between ≤2.0 and ≤2.5) ◦ Abnormal coronary microvascular resistance indices (e.g., IMR > 25) ◦ Coronary microvascular spasm, defined as reproduction of symptoms, and ischemic ECG shifts, but no epicardial spasm during acetylcholine testing ◦ Coronary slow-flow phenomenon, defined as a TIMI frame count > 25	**Nitrate-responsive angina**, with at least one of the following: ◦ Rest angina ◦ Marked diurnal variation in exercise tolerance ◦ Hyperventilation can precipitate an episode ◦ Calcium channel blockers (but not β-blockers) suppress episodes **Transient ischemic ECG changes**, including any of the following in at least two contiguous leads: ◦ ST segment elevation ≥ 0.1 mV ◦ ST segment depression ≥ 0.1 mV ◦ New negative U waves **Coronary artery spasm**, defined as transient total, or subtotal, coronary artery occlusion (>90% constriction), with angina and ischemic ECG changes, either spontaneously, or in response to a provocative stimulus (typically acetylcholine ergot, or hyperventilation)

CAD: coronary artery disease; FFR: fractional flow reserve; CTA: computed tomography angiography; ECG: electrocardiogram; IMR: index of microvascular resistance; TIMI: thrombolysis in myocardial infarction.

**Table 3:** Invasive methods of coronary microvascular function assessment.

Index	Principle	Equation	Advantages	Disadvantages
Coronary Flow Reserve (CFR)	Thermodilution	$\frac{Tmn\;rest}{Tmn\;hyper}$	-Feasible -Safe -Reproducible	-Requires hyperemia -Operator-dependent
	Doppler	$\frac{CFVhyper}{CFV\;rest}$		
Index of Microcirculatory Resistance (IMR)	Thermodilution	*Pd* × *Tmn_[hyper]_*	-Feasible -Safe -Reproducible	-Requires hyperemia -Operator-dependent -May overestimate true microvascular resistance with critical epicardial CAD
Hyperemic Microvascular Resistance (HMR)	Doppler	$\frac{Pd}{APV\left[hyper\right]}$	-Feasible -Safe -Reproducible	-Requires hyperemia -May overestimate true microvascular resistance with critical epicardial CAD
Minimal Microvascular Resistance (mMR)	Doppler	$\frac{Pd}{Qs\left[wf\;period+hyper\right]}$	-Feasible -Safe -Reproducible -Good correlation with true microvascular resistance, irrespective of any epicardial CAD	
Resistive Reserve Ratio (RRR)	Thermodilution	$\frac{BIR}{IMR}$	-Measure of vasodilation capacity of coronary microcirculation	-Requires hyperemia -Operator-dependent
Absolute Coronary Flow and Resistance	Continuous thermodilution		-Safe -Reproducible -Operator-independent -Pharmacological-hyperemia not required	-Standardized reference values not available

Tmn: mean transit time; CFV: coronary flow velocity; Pd: distal coronary pressure; APV: average peak velocity; Qs[wf period+hyper]: flow velocity during the hyperemic wave-free period; BIR: basal microcirculatory resistance; CAD: coronary artery disease.

## Data Availability

No new data were created or analyzed in this study. Data sharing is not applicable to this article.
